# The gendered context of women charged with violent offences in the forensic psychiatric setting

**DOI:** 10.4102/sajpsychiatry.v30i0.2222

**Published:** 2024-03-30

**Authors:** Mohammed Nagdee, Lillian Artz, Ugasvaree Subramaney, Charles Young, Amanda Pieterse, Julia Pettitt

**Affiliations:** 1 Department of Psychological Medicine, Faculty of Health Sciences, University of Otago, Dunedin, New Zealand; 2 Gender, Health and Justice Research Unit, Faculty of Health Sciences, University of Cape Town, Cape Town, South Africa; 3 Department of Psychiatry, Faculty of Health Sciences, University of the Witwatersrand, Johannesburg, South Africa; 4 School of Psychology, College of Health and Education, Murdoch University, Perth, Australia

**Keywords:** female offenders, women offenders, violent offenders, forensic, mental health, South Africa

## Abstract

**Background:**

Women charged with violent offences may be referred by courts for forensic psychiatric assessment to determine whether mental disorder or intellectual disability impacts their fitness to stand trial and/or criminal responsibility. The profile of these women is a poorly researched area in South Africa.

**Aim:**

This study examined the socio-demographic, offence-related, and clinical profile of South African women charged with violent offences referred for forensic assessment.

**Setting:**

Fort England Hospital (FEH), a forensic psychiatric institution in the Eastern Cape.

**Methods:**

The clinical records of 173 women referred by courts for forensic psychiatric evaluation over a 24-year period (1993–2017) to FEH were systematically reviewed.

**Results:**

Most women were single, black mothers with dependent children, who were unemployed and socio-economically impoverished. Many had backgrounds of pre-offence mental illness, alcohol use and alleged abuse. The majority were first-time offenders whose victims were known to them. Most child victims were biological children killed by their mothers. Likely primary motives for violence were related to psychopathology in half of cases, and interpersonal conflict in a third. Forensic assessment most frequently confirmed psychotic disorders and dual diagnoses. Half the cases were fit to stand trial and under half were criminally responsible.

**Conclusion:**

Violent female offending occurs within a gendered context, with high rates of prior trauma, alcohol use and psychosocial distress in perpetrators. An emphasis on gender-sensitive psychosocial interventions is required.

**Contribution:**

This study highlights the nature and context of violent offending by women referred for forensic psychiatric assessment in South Africa.

## Introduction

The mental health of female offenders has not been as well researched as that of male counterparts. In South Africa, women’s criminal pathways typically feature backgrounds of physical and sexual trauma, familial neglect and conflict, significant psychosocial stressors, substance use and mental health problems.^1,2,3,4,5^ International research highlights numerous criminogenic factors associated with offending by women, including early behavioural problems, family and social instability, poor education, unemployment, single motherhood, substance use and financial deprivation.^6,7^ The literature also reveals that imprisoned women are up to twice as likely as imprisoned men to have mental disorder, presenting most commonly with self-harm, suicidality, substance use, depression, anxiety and trauma-related disorders, and personality disorders.^8,9,10,11,12^

While female-perpetrated violence has grown in recent decades, most violent offending is still committed by men, although this gender difference may be less pronounced in the context of severe mental disorder.^13,14,15^ There is relatively little published data on the antecedents to mental disorder *and* violent offending in women, especially in developing countries. This may be partly because of antiquated perceptions about female aggression, especially in the many societies within which traditionally patriarchal views on gender roles persist. While violent women with mental health problems more commonly carry out non-lethal offences, most research has focused on lethal forms of female violence.^16^ Although severe mental disorder, especially schizophrenia, is associated with an increased risk of violent behaviour irrespective of the gender of perpetrators, this association has been postulated to potentially be more robust in women.^17,18,19^ Studies on violent women in forensic psychiatric settings are diverse in their findings, but consistently report high rates of schizophrenia and other psychoses, depressive disorders, substance-related disorders and personality disorders.^19,20,21^

Studies on forensic psychiatric samples in South Africa have primarily examined male offenders.^22,23,24,25,26,27^ Nonetheless, a few studies have focused on psychosocial, criminogenic and clinical factors associated with female offenders. An early study indicated that both ethnicity and gender may affect the process of court referrals.^28^ Another study described the mental health status of 32 women charged with lethal violence towards children who were referred for psychiatric evaluation.^29^ While over 40% of women had no psychiatric diagnosis, psychotic and mood disorders comprised most cases who were mentally ill. The majority of women were found to be fit to stand trial and criminally responsible for their offences. A subsequent multi-site collaborative project study documented the clinical and psycho-social profile of 573 South African women referred for forensic psychiatric assessment and highlighted that most women were from impoverished communities, were often exposed to trauma themselves and had high pre-offence rates of mental disorder and alcohol use.^5^ The majority of women in this survey were charged with violent offences, most commonly murder, directed at victims well known to them. High rates of psychotic and mood disorders were reported, although most defendants were found to be fit to stand trial and criminally responsible for their alleged offence. More recently, a survey of 126 female inmates in KwaZulu-Natal found that almost two-thirds of study participants were human immunodeficiency virus (HIV)-positive and had disproportionately high lifetime prevalence rates of mental disorder, especially depression, psychosis, substance misuse, posttraumatic stress disorder (PTSD), personality disorders and hyperactivity disorder (ADHD).^10^

## Aims

This study sought to systematically examine the hospital records of women charged with violent offences who were court-referred forensic psychiatric assessment to Fort England Hospital (FEH), Eastern Cape, in respect of their socio-demographic, offence-related, and forensic psychiatric profile.

## Research methods and design

Purposive sampling of all 173 women who were court-referred to FEH for formal forensic evaluation under the *Criminal Procedure Act* 51 of 1977, as amended (CPA) between 1993 and 2017 was conducted. Retrospective, systematic examination of the criminal, administrative and forensic psychiatric records of all cases was conducted via a structured, standardised data-collection sheet. A range of socio-demographic, offence-related and forensic clinical data were collected. Fort England Hospital was the primary forensic psychiatric referral facility for the Eastern Cape, and conducted the vast majority of all forensic psychiatric assessments in the province and all those involving serious, violent offences in the review period (a small number of assessments were conducted at Komani Hospital, although only single-psychiatrist observations for non-violent offences).^30^ Descriptive data analysis was carried out using SPSS (version 22.0.0.0).

### Ethical considerations

Data extracted from clinical hospital records were deidentified in the interests of anonymity and confidentiality, with each case being assigned a unique study participation number. The physical and digital study data were securely kept by the primary researcher in a site with biometric access control. Formal ethics clearance was obtained from the Rhodes University Research Projects and Ethics Review Committee. Written approval to commence the study was subsequently obtained from the Eastern Cape Department of Health: Epidemiological Research and Surveillance Management Directorate, and the Hospital Manager of FEH.

## Results

Forensic psychiatric assessments were conducted on 173 women at FEH between 1993 and 2017, and the archival records of all these cases were systematically examined regarding a range of socio-demographic, offence-related and forensic variables.

### Offence profile

Offences were classified using Snyman’s classification of offences for South Africa, which includes crimes against: (1) the state; (2) the community; (3) the person (e.g. attempted murder, murder, assault and assault with intent to do grievous bodily harm) and (4) property, respectively.^31^

Most women (80%) were facing their first criminal charge(s) when referred for forensic assessment. For the 20% of women with prior convictions, most related to non-violent crimes (56% of these cases). Among the remaining minority with prior violent offending histories (44% of cases), non-lethal assaultive violence comprised approximately 40%, with less than 5% having committed prior offences against life. Furthermore, over 60% of women charged with offences against life (attempted murder and murder) had a confirmed prior criminal record.

Table 1 summarises the index offence profile of the study sample (*n* = 173). All crimes against a person (*n* = 100) and sexual crimes (*n* = 1) were considered violent index offences, accounting for the majority of offences (58%). Over half of all index offences were accounted for by just three specific violent crimes: assault with intent to do grievous bodily harm, attempted murder, and murder, respectively.

**TABLE 1:** Index offence profile (*N* = 173).†

Offence category	Offence sub-category	Index offence	Frequency	%
Crimes against the community (*n* = 10)	Sexual crimes	Sexual assault	1	0.6
	Crimes against the family	Contravention of protection order	4	2.3
	Crimes against public welfare	Concealment of birth	1	0.6
		Discharging a firearm	1	0.6
		Drug offences	3	1.7
Crimes against a person (*n* = 100)	Crimes against life	Attempted murder	6	3.5
		Murder	45	26.0
	Crimes against bodily integrity	Assault	6	3.5
		Assault with intent to do grievous bodily harm	36	20.8
		Domestic violence	4	2.3
		Intimidation	1	0.6
	Crimes against dignity and reputation	Criminal defamation	1	0.6
	Crimes against freedom of movement	Kidnapping	1	0.6
Crimes against property (*n* = 63)	Crimes related to appropriation of property	Robbery	3	1.7
		Theft	23	13.3
	Fraud and related crimes	Fraud	7	4.0
	Crimes related to damage to property	Arson	4	2.3
		Housebreaking with intent and related crimes	4	2.3
		Malicious injury to property	22	12.7

**Total**			**173**	**100**

† , Categorised using an adaptation of Snyman’s (2014) classification.

### Forensic psychiatric profile

The key socio-demographic, offence-related and forensic psychiatric features of women charged with violent offences are summarised in Table 2. The mean age was 37.8 years. The majority of defendants were black people, single mothers with dependent children who were unemployed at the time of their arrest. Most women had no prior criminal history. Most women had documented pre-offence psychiatric histories (69%) and/or backgrounds of substance use (55%). While a history of pre-offence abuse of women themselves was unknown or unspecified in the majority of case records (67%), prior abuse (physical, sexual, emotional or other) was reported by 33 women (33%). The relationship to alleged abuse perpetrators was documented in 90% of these cases, with the majority of these occurring within the domestic or family context, especially by intimate male partners, parents and other close relatives.

**TABLE 2:** Forensic psychiatric profile of violent offenders (*N* = 101).

Variable	Frequency	%
**Socio-demographic**		
Age over 30 years	67	66.3
Black people	85	84.2
Single	73	72.3
Dependent children	89	88.1
Highest level of education > Grade 8	56	55.4
Unemployed at arrest	84	83.2
**Offence-related**		
Prior criminal history	34	33.6
Weapon use for offence	52	51.5
*Likely primary motive for offence:*		
Psychopathology	53	52.5
Interpersonal conflict (in absence of psychopathology)	34	33.7
**Clinical background**		
Prior abuse of offender (self-reported)	33	32.7
Psychiatric history	70	69.3
Substance use history	55	54.5
Severe mental disorder	71	70.3
**Forensic outcomes**		
Fit to stand trial	49	48.5
Criminal responsibility	44	43.6

In respect of the nature of violent index offences, a weapon was employed in half of cases, most commonly a knife or other sharp object was used to stab victims, followed by use of a blunt object to inflict injury, battery without weapons, and strangulation or suffocation, respectively. While almost one-third of attempted murder or murder cases involved stabbing, there were no cases of firearm usage to lethal or near-lethal effect.

Forensic psychiatric assessment confirmed the presence of severe mental disorder in over two-thirds of cases (*N* = 71; 70%). Psychotic disorders comprised the most common single diagnostic category, being present in almost one-third of all cases (*N* = 32; 32%), with schizophrenia being the single most common psychiatric diagnosis (*N* = 19; 19%). Over one-third of women (*N* = 34; 34%) had a substance-related diagnosis, either independently or in combination with another mental disorder, with alcohol being implicated more than all other substances combined. Almost one-quarter of women were documented to have been intoxicated with alcohol at the time of the alleged offence. Active psychopathology was considered the likely primary motive for offending behaviour in over half of cases. In the remainder, likely primary motives related to interpersonal conflict accounted for almost a third. Half of the women (49%) were considered fit to stand trial and 44% were deemed criminally responsible for their actions at the time of alleged offences. Forty-one women (41%) were deemed to be both fit to stand trial and criminally responsible. Almost half of women (*N* = 49; 49%) were found to be both unfit to stand trial and criminally responsible for their offences.

### Victims of violence

Information on age of victims was specified in the clinical records of 101 cases, the majority of whom (*N* = 69) were adults (Table 3). Adult women comprised almost two-thirds of victims, with most of these (in excess of 90%) being known to the alleged perpetrator: intimate male partners (19%), family members (35%) and neighbours, friends or acquaintances (30%), respectively. Likely primary motives for violence against adults, as documented in the clinical record by forensic assessors, were varied: active psychopathology in 58% of cases, followed by violence related to interpersonal conflict, in the absence of active psychopathology, in a further one-third (the latter mostly towards those well known to them in the domestic, family or social setting).

**TABLE 3:** Violence against adults (*N* = 69).

Variable	Frequency	%
**Gender of victim**		
Male	24	34.8
Female	42	60.9
Unknown	3	4.3
**Relationship of victim to offender**		
Intimate male partner	13	18.8
Parent	9	13.0
Sibling	5	7.3
Other relatives	10	14.5
Neighbour, friend or acquaintance	21	30.4
Stranger	4	5.8
Unknown	7	10.2
**Likely primary motive for offence**		
Psychopathology	40	58.0
Interpersonal conflict in absence of psychopathology	23	33.3
Other or unknown	6	8.7

Children comprised approximately one-third of victims (*N* = 32), with the majority of these (88%) having been murdered. Table 4 summarises data concerning the age and gender of victims, relationship to offenders and likely primary offending motives against children.

**TABLE 4:** Violence against children (*N* = 32).

Variable	Frequency	%
**Age of victims**		
Neonate (< 1 month)	9	28.1
Infant (1 month – 1 year)	8	25.0
1–5 years old	8	25.0
Greater than 5 years old	7	21.9
**Gender of victims**		
Male	14	43.8
Female	13	40.6
Unknown	5	15.6
**Relationship with offender**		
Biological child	26	81.3
Child of family member	3	9.4
Child of friend or acquaintance	1	3.1
Child of stranger	2	6.2
**Likely primary motive for offence**		
Psychopathology	13	40.6
Interpersonal conflict (in absence of psychopathology)	6	18.8
Unwanted child	5	15.7
Impulsive	4	12.5
Substance-related	2	6.2
‘Altruistic’	1	3.1
Unknown	1	3.1

The mean age of child victims was 30.3 months. Over 53% of children were less than a year old, with the risk being disproportionately high for neonates and reducing with increasing age of victims. In the 27 cases in which the gender of victims was known, approximately equal proportion of males and females were present. Biological children comprised the majority (81%) of cases and child victims were known to the offenders in almost 95% of instances (Table 4).

Likely primary motives related to active psychopathology at the time of offence were documented in over 40% of cases (compared to 58% for adult victims – see Table 3). Interpersonal conflict, in the absence of active psychopathology and most commonly with the fathers of victims, was reported in almost one-fifth of cases. Children who were perceived as unwanted accounted for 16% of cases, with 13% being attributed to impulsive violence following perceived provocation by the child victim. Violence related to substances (most commonly because of alcohol intoxication or withdrawal) was documented in 6% of cases, with a solitary case of ‘altruistic’ violence carried out in an attempt to relieve extreme socio-economic distress.

## Discussion

### Offence profile

Most of the women in this study had no prior criminal convictions, a pattern consistent with other studies, both nationally and abroad.^2,5,8,9,12,32^ In comparison to women without previous convictions, for women with prior criminal records, the most previous offence was for non-violent, acquisitive crimes while alleged index offences were more likely to have been of violence, again confirming published trends in other forensic surveys.^19^

Unlike similar studies in forensic settings abroad, the vast majority of index offence charges in the present study sample were of a violent nature with murder being the most common single offence.^32,33,34,35^ Furthermore, the majority of violent women in studies abroad commit less severe assaultive crimes (i.e. ‘simple or common’ as opposed to ‘aggravated’ assault).^11,36^ In contrast, most women in the current study who were accused of committing non-lethal violence were charged with the more severe assault with intent to do grievous bodily harm. Half of the women sampled also used weapons to commit violent acts, in contrast to the minority of women in other surveys who do so.^36^

The reasons for the disproportionately violent index offence profile in the Eastern Cape are complex and likely to be related to the broader, multidimensional aetiology of violence in South African society. The socio-demographic characteristics of the study sample are very similar to the broader population of the Eastern Cape: a province that is historically and significantly under-resourced and impoverished.^37^ Important contextual factors would hence include: the persistently deleterious consequences of apartheid; socioeconomic adversity and inequality; family instability; poor educational, economic and occupational opportunities; a lack of social cohesion; weapon access; substance use; growing proportion of marginalised, unemployed and poorly supported youth; and systemic failures within criminal justice, health and social welfare systems; and so on.^38,39^ These criminogenic variables cannot be isolated from the gendered context within which they are embedded, especially the influence of gender norms, roles, stereotypes, identity, sexuality, controls and stigma, and uniquely individual responses to these.^33,34,40^ The profile of the violent women in the study sample should also be seen as a likely product of this psychosocial milieu, being largely a cohort of unemployed, impoverished, black, single mothers from the Eastern Cape. This is compounded in many cases by backgrounds of prior abuse, substance use and/or mental health problems, which potentially further increase vulnerability to worsening mental health and violent behaviour.^11,34,41^

A significant proportion of violent index offences in the study also took place in the context of interpersonal conflict with people well known to defendants. In many cases, this resulted in reportedly self-defensive or retaliatory offences towards intimate male partners, family members or victims within close social circles. This emphasises the important criminogenic potential of domestic and social dynamics in respect of violent female offfence.^33,35,42^ Women who experience distress within systems of socio-cultural inequality may well be more prone to responding violently, although many confounding factors prevent direct causal conclusions. Family and social discord, and the associated psychological distress, are postulated to contribute to mental health problems in many women, further increasing their vulnerability to potentially violent behavioural outcomes. This complex interplay of multiple, dynamic psychosocial variables that are both gendered and uniquely individual, is postulated to underlie the pathways to violent offending of many South African women.

### Forensic and mental health issues

The socio-demographic profile of the study sample was consistent with other research trends that violent women, in comparison to both violent men and non-violent women in forensic settings, tend to be slightly older, single mothers with dependent children, who are socioeconomically deprived and have relatively poor educational and occupational attainment.^33,43,44^ Violent women are also more likely to have experienced prior trauma, misuse substances and suffer from mental health problems.^8,9,33,43,45,46,47,48,49,50^ Early trauma is not only associated with the risk of abused girls becoming violent women themselves, but to the emergence of psychological distress and mental disorder.^16,46^ The risk of committing violence by women who were previously abused is known to often occur in the context of family, relational and social instability, compounded by the distress and vulnerability that these women experience throughout their lives.^41,45^ Early trauma exposure has been associated with maladaptive patterns of processing social information, with this being an additional variable potentially mediating the emergence of future aggression.^46^ Associations between historical abuse and future violence were similarly apparent in this study. In the study sample, almost one-third of women had pre-offence backgrounds of being abused themselves. Furthermore, while 15% of women facing non-violent index offences reported prior abuse, this was the case in over 30% of women charged with violent index offences.

While the vast majority of violent offences are perpetrated by men, violent offence by women is increasing globally with this gender gap being potentially further reduced in cases of offenders with more severe forms of mental disorder.^34,36,51^ Unsurprisingly for a forensic study sample, relatively high rates of severe mental disorder were present, with psychotic-spectrum disorders (especially schizophrenia) being the most common diagnostic category. The risk of violence by people with psychosis is associated with acuteness and/or severity of psychotic symptoms (persecutory delusions and command hallucinations in particular), threat-control override, comorbidity, and antipsychotic non-adherence.^14,47,52^ Psychosis may also confer a disproportionate risk for violent behaviour in women as compared to men, for reasons that are not entirely clear.^18,51,53^ Women who suffer from psychotic disorders, however, also tend to have shorter criminal careers and desist from violent offence sooner than either violent men with psychosis or violent women without psychosis.^51^

The study also confirmed relatively high rates of prior substance use and substance-related disorders, often comorbid with diagnoses of severe mental disorder. Women who misuse substances, especially alcohol, are known to be at increased risk of violence (including recidivistic violence) although the nature of this association is complex and the extent of it unclear.^18,34,36^ Alcohol misuse by women within criminal justice and forensic settings is also associated with acts of violence against intimate partners in particular, a pattern confirmed in this study.^54^ Comorbid substance misuse by women who suffer from psychosis has also been shown to further increase the likelihood of violence being directly driven by psychotic symptoms such as delusional beliefs.^51^ The relationship between severe mental disorder and violent outcomes may be mediated in many women by the presence of psychiatric comorbidity especially in the form of dual diagnoses.^17^

The presence of severe mental disorder in women is hence likely to be a vital, although not necessarily sufficient, mediating factor leading to violent offence. On the one hand, many gender-neutral features of severe mental disorder contribute to the risk of violence in general, including, for example, impaired reality testing, impulse dyscontrol, poor affective regulation, cognitive inflexibility, distorted social judgement, aberrant salience attribution, threat-control override, disorganised thinking and behaviour, and executive dysfunction. Violent women who experience severe form of mental disorder need to be understood by integration of their psychopathological features with an appreciation of the unique psychological, social, interpersonal and environmental context within which such violence is embedded.^51,52^ While severe mental disorder is likely to modify how gender may influence the risk of behaving violently, it remains unclear how, and to what extent, this is likely to occur. Stueve and Link posed a few key questions in this respect, including whether: (1) the subjective experience of severe mental disorder in some women may alter cognition, perception, affect and behaviour to such an extent that mechanisms that would otherwise deter violence are either less effective or no longer operative; (2) others may respond differently (e.g. more slowly or less effectively) towards women with severe mental disorder in their efforts to defuse potentially violence situations; and (3) women with severe mental disorder may, as a result of the nature and/or severity of their psychiatric symptoms, have impaired ability to cope with distressing situations or traumatic events.^55^ The importance of additional contextual factors has also been highlighted in the research literature including intense personal and family discord (especially with intimate partners and close family members), social distress; economic and occupational marginalisation; entrenched gender roles, especially within patriarchal settings; and early exposure to trauma.^48,49,56^

### Victims of violence

In excess of 90% of violence by women in the study sample was directed towards victims within immediate family or inner social circles, confirming well established trends from other studies.^5,16,36,43,44,50,57^ Motives for violent behaviour in women directed at those close to them are postulated to be typically driven by intense personal conflict often, but not exclusively, in the context of self-defence under threat, defence of moral or sexual virtue, or impulsive behavioural responses to provocation, abuse or violence from others.^34^ In the presence of severe mental disorder, however, violence may also be related to and/or mediated by the unique features of the woman’s mental state at the time of the event. The risk of violence by women towards intimate male partners and close family members in particular is associated with a history of their own prior abuse at the hands of their victims.^16,42,49,57^ Not only is early-onset trauma an independent predictor for later violence by such women, but trauma-mediated distress is also linked to the development of subsequent mental disorder.^45,46,48,49^

Children comprised almost a third of all victims in this study, with the risk of children falling victim of violence being highest in neonates, followed by infants, consistent with findings of other studies both locally and abroad.^5,11^ Biological children in their first year of life were particularly vulnerable, especially when maternal perpetrators of violence were suffering from severe mental disorder, as has been documented elsewhere.^5,11,58^ Likely primary motives for violence towards children were also diverse, and included offences related to psychopathology, interpersonal conflict, maternal care-giving, substance intoxication, and so-called ‘altruistic’ acts respectively.

## Conclusions

This study has several limitations, including its retrospective, descriptive nature and the absence of male offender comparative data. The retrospective study design inevitably produces limitations regarding generalisability, data accuracy, missing or unknown information in some cases, and possible sources of bias (case-inclusion and information bias in particular). Nonetheless, some important conclusions can be drawn regarding women charged with violent offences in the South African forensic psychiatric setting. Most women were single, black mothers with dependent children, with relatively low levels of education who were unemployed and socio-economically impoverished. Many had backgrounds of pre-offence mental illness, alcohol use and alleged abuse. The majority were first-time offenders charged with violent crimes against the person, with murder being the most common index offence. It is proposed that the phenomenon of such violence needs to be understood within its distinctly gendered psychosocial context. Pathways to such violence can only be meaningfully addressed if, in addition to psychiatric treatment and management of risk, a range of gender-based psychosocial and systemic interventions are pursued. Further research exploring the complex interplay of contextual criminogenic factors is required on female forensic populations, especially in developing countries such as South Africa, with a particular focus on gender-related risk predictors and mediators of violence. Furthermore, studies that include both male and female forensic samples would generate more meaningful gender-focused comparisons.
